# The patient is still there: geriatric psychiatry and the legacy of deinstitutionalization

**DOI:** 10.1093/geroni/igag086

**Published:** 2026-07-22

**Authors:** James V English, Jess Malouf, Callie Ward

**Affiliations:** Missoula, Montana, United States; School of Physical Therapy and Rehabilitation Science, University of Montana, Missoula, Montana, United States; Occupational Therapy Graduate Program, School of Medicine, University of New Mexico, Albuquerque, New Mexico, United States

**Keywords:** Discharge planning, Long-term care capacity, Care transitions, Dementia care, Health policy

Older adults with severe psychiatric illness can remain hospitalized after acute treatment has done what it can (Salime et al., 2022). Too often, that continued stay is read as failed discharge planning or incomplete treatment, when it reflects failure in the system meant to receive that patient. Geriatric psychiatric patients are not failing to be discharged. They are failing to have anywhere to go. The first is a clinical problem with a clinical solution. The second is structural. Stabilization is not the same as placement. Stabilization means acute safety and a response to medication. It does not mean the behavioral, cognitive, and medical burden is gone. The bottleneck lies less in whether the patient can leave the hospital than in whether any receiving setting can take that patient safely. This account is American by design. Other aging societies face the same pressure. They have more older adults and too few places to send them. But the particular machinery here is national: the statutes, the exclusions, the missing registries. Other countries share the predicament. They do not share the laws that made it.

That distinction is especially important for older adults with dementia complicated by psychiatric, behavioral, and medical burden. Their symptom burden and behavioral complexity often exceed what many skilled nursing and assisted living settings can manage. This is not simply a matter of diagnosis. It is a problem of fit, but also of receiving-system incapacity. Barriers to discharge in this population include behavioral symptoms, limited psychiatric treatment access, insurance barriers, weak social support, and waiting lists. The obstacle is often not continued need for acute psychiatry, but the absence of any downstream setting willing to absorb the burden that remains (Hoffman et al., 2025; Kales et al., 2015; Toot et al., 2017).

This bottleneck has a history. The Community Mental Health Act of 1963 promised that institutional care would be replaced by community systems (Mental Retardation Facilities and Community Mental Health Centers Construction Act of 1963). That promise was never fully realized (Salime et al., 2022). Some patients were easier for the downstream system to absorb than others. Older adults with the greatest behavioral, cognitive, and medical complexity were not. What followed was a slow accumulation of abandonments, each one defensible on its own. Together they were catastrophic. Institutions emptied faster than the system built to replace them. For older adults with severe mental illness, dementia, and layered comorbidity, the result has been a late-life care continuum too thin, fragmented, and opaque to receive them reliably (Salime et al., 2022). The discharge team did not build this. They inherited it.

The exclusion was built on purpose. When Medicaid was created in 1965, Congress wrote in the Institutions for Mental Diseases exclusion, barring federal payment for most care in large psychiatric facilities (Social Security Amendments of 1965). The aim was to end warehousing and move care into the community. The reasoning addressed the institutions. It did not follow the patient who still needed somewhere to go. The same pattern repeated decades later. When federal incentives drove hospitals to adopt electronic records, psychiatric facilities were left outside the effort, partly to protect sensitive records under separate confidentiality law (Office of the National Coordinator for Health Information Technology, 2022). Each step had its reasons. None planned for the patient who would still be waiting.

That is why this problem belongs to aging policy and practice, not only to hospital psychiatry. Discharge teams are asked to make some of the most consequential placement decisions with less public quality information than exists elsewhere in late-life care. It sits at the intersection of dementia care, long-term care capacity, care transitions, and family burden. The patient who remains hospitalized marks more than a discharge delay. The patient reveals a failure of integration across the care continuum. What looks like a hospital problem is also a long-term care problem, a dementia-care problem, and a systems problem for aging itself (National Academies of Sciences, Engineering, and Medicine, 2021).

Three changes would begin to address it, none of them novel. Each extends a standard already expected elsewhere in health care. Psychiatric hospitals need a public quality registry that makes oversight findings visible. Receiving facilities should be required to disclose admission criteria in a form discharge teams and families can use. When a facility declines a geriatric psychiatric patient, that refusal may reflect selective admission incentives yet stay invisible to discharge teams and families. And psychiatric hospitals should be brought into modern health information systems, not left outside them (Office of the National Coordinator for Health Information Technology, 2022). Federal discharge rules already require attention to the fit between patient needs and environmental capacity. Psychiatric hospitals still lack the information to meet it (42 C.F.R. § 482.43; Office of the National Coordinator for Health Information Technology, 2022). What cannot be seen cannot be fixed. The patient is still there: the human evidence of what reform promised but never built.

## Data Availability

No new data were generated or analyzed in support of this manuscript.
